# Elderly woman with soft tissue ossification

**DOI:** 10.11604/pamj.2017.28.307.14507

**Published:** 2017-12-13

**Authors:** Rodolfo Mendes Queiroz, Fred Bernardes Filho

**Affiliations:** 1 Department of Radiology and Imaging, Santa Casa da Misericórdia of Avaré, Avaré, São Paulo, Brazil; 2 Centromed Diagnóstico por Imagem, Avaré, São Paulo, Brazil; 3 Dermatology Division, Department of Medical Clinics, Ribeirão Preto Medical School, University of São Paulo, Ribeirão Preto, Brazil

**Keywords:** Calcinosis, venous insufficiency, osteogenesis

## Image in medicine

A 67-year-old woman with hypertension, type 2 diabetes and chronic venous insufficiency (CVI) was admitted to the emergency room due painful left leg after a fall from own height. The fall had been a true mechanical one, with no preceding dizziness, chest pain, or palpitations. On physical examination, small chronic ulcer and subcutaneous white calcifications were observed. Hemogram, electrolytes, cardiac enzymes, parathyroid hormone, calcium, phosphorus, serum creatinine and 25-hydroxycholecalciferol were unremarkable. A plain radiograph of her left leg showed no fracture and multiple radio-opaque images, clearly outlined in the subcutaneous tissues. The diagnosis of dystrophic subcutaneous calcification was made. The patient refused clinical follow-up. Calcinosis cutis (CC) is defined as the deposition of calcium salts in the skin. The condition is divided into 5 types: calciphylaxis and dystrophic, metastatic, idiopathic and iatrogenic CC. Dystrophic calcification is the most frequent type of CC. Serum calcium and phosphorus levels are normal. Local tissue damage, such as degeneration of collagen fibres, elastic fibres, fat cell necrosis caused by trauma, infections, neoplasms and many other conditions, are considered to be precipitating factors. Calcium deposits impair normal epithelial growth and cause persistent inflammation with a foreign body reaction, resulting in delayed wound healing. Dystrophic calcification is a major cause of failure of conservative ulcer management and it is considered a challenge in CVI patients.

**Figure 1:**
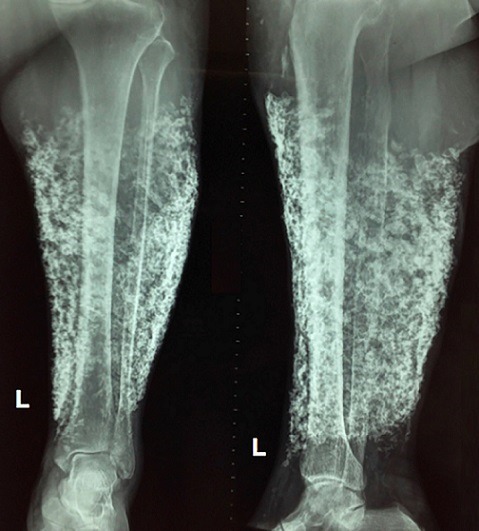
Radiograph of the left leg, demonstrating severe subcutaneous dystrophic calcification

